# Bipolar depression: a major unsolved challenge

**DOI:** 10.1186/s40345-019-0160-1

**Published:** 2020-01-06

**Authors:** Ross J. Baldessarini, Gustavo H. Vázquez, Leonardo Tondo

**Affiliations:** 1 Department of Psychiatry, Harvard Medical School, Boston, MA USA; 2 International Consortium for Bipolar & Psychotic Disorders Research, McLean Hospital, Belmont, MA USA; 3 Department of Psychiatry, Queen’s University School of Medicine, Kingston, ON Canada; 4 Lucio Bini Mood Disorder Center, Cagliari, Sardinia Italy

**Keywords:** Bipolar disorder, Depression, Disability, Morbidity, Mortality, Suicide, Treatment

## Abstract

Depression in bipolar disorder (BD) patients presents major clinical challenges. As the predominant psychopathology even in treated BD, depression is associated not only with excess morbidity, but also mortality from co-occurring general-medical disorders and high suicide risk. In BD, risks for medical disorders including diabetes or metabolic syndrome, and cardiovascular disorders, and associated mortality rates are several-times above those for the general population or with other psychiatric disorders. The SMR for suicide with BD reaches 20-times above general-population rates, and exceeds rates with other major psychiatric disorders. In BD, suicide is strongly associated with mixed (agitated-dysphoric) and depressive phases, time depressed, and hospitalization. Lithium may reduce suicide risk in BD; clozapine and ketamine require further testing. Treatment of bipolar depression is far less well investigated than unipolar depression, particularly for long-term prophylaxis. Short-term efficacy of antidepressants for bipolar depression remains controversial and they risk clinical worsening, especially in mixed states and with rapid-cycling. Evidence of efficacy of lithium and anticonvulsants for bipolar depression is very limited; lamotrigine has long-term benefit, but valproate and carbamazepine are inadequately tested and carry high teratogenic risks. Evidence is emerging of short-term efficacy of several modern antipsychotics (including cariprazine, lurasidone, olanzapine-fluoxetine, and quetiapine) for bipolar depression, including with mixed features, though they risk adverse metabolic and neurological effects.

## Background: depression in bipolar disorder

### Nosological uncertainties

Debate concerning Kraepelin’s broadly inclusive concept of manic-depressive illness (MDI) continued to 1980 with a first formal separation of a distinct *bipolar disorder* (BD) with mania from nonbipolar major depressive disorder (MDD) in DSM-III (Trede et al. 2005; Baldessarini et al. 2015). Tension continues between lumping mood syndromes and separating various depressive and bipolar subtypes, and considering a “spectrum” of disorders ranging from more or less pure depression to archetypical BD, leading to profound therapeutic ambiguities (Cuellar et al. 2005; Goodwin and Jamison 2007; Baldessarini 2013; Yildiz et al. 2015; Tondo et al. 2018).

### Current status of bipolar depression

Adequate understanding, timely diagnosis, and effective short- and long-term treatment of depressive episodes in BD patients are critically important but remarkably insufficiently resolved (Baldessarini et al. 2010c). Clinical significance of bipolar depression is underscored by strong association with overall morbidity, other co-occurring psychiatric conditions (notably anxiety and substance-abuse disorders), disability, and excess mortality owing largely to suicide in young patients and intercurrent medical illness in older patients (Ösby et al. 2001, 2018; Tondo et al. 2014, 2016; Baldessarini et al. 2020).

### Diagnosis

Clinical challenges include difficult and often long-delayed diagnostic differentiation of depression as an initial presentation of BD vs. a manifestation of nonbipolar MDD. Accurate diagnosis and appropriate treatment typically are delayed by 6–8 years, and even longer following juvenile onset (Post et al. 2010; Bschor et al. 2012; Drancourt et al. 2013; Tondo et al. 2014). Depression is initially considered as unipolar MDD in as many as 40% of patients later diagnosed with BD (Stensland et al. 2008; Shen et al. 2018). Such uncertainty is heightened as depression is the most prevalent presenting polarity in BD, (Goodwin and Jamison 2007; Baldessarini et al. 2014; Yildiz et al. 2015). Moreover, excess future depression in BD can be anticipated by initial episodes of anxiety or mixed-states as well as of depression (Baldessarini et al. 2012, 2014, 2020).

BD patients commonly fear, seek to avoid, to report, and to seek clinical help for depression. Contrarily, they may not recognize moderate increases of mood, energy, activity, or libido as hypomanic symptoms as clinically relevant, and may even prefer such states. Diagnostic uncertainty is especially likely early in the illness-course and without corroborating information from a family member or close friend (Vöhringer and Perlis 2016).

In perhaps 12–17% of cases, BD is not recognized until there is a mood “switch” into hypomania or mania (“[hypo]mania”), either spontaneously or with exposure to a mood-elevating substance (Tondo et al. 2010; Baldessarini et al. 2013; Barbuti et al. 2017). Other indirect factors suggesting a diagnosis of BD include: (a) familial mania, psychosis, “nervous breakdown,” or psychiatric hospitalization; (b) early illness-onset, commonly with depression; (c) cyclothymic temperament; (d) multiple recurrences (e.g., ≥ 4 depressive episodes within 10 years); (e) depression with prominent agitation, anger, insomnia, irritability, talkativeness, other “mixed” or hypomanic features, or psychotic symptoms; (f) clinical “worsening,” especially with mixed features during an antidepressant treatment; (g) suicidal ideation and acts; and (h) substance abuse (Tondo et al. 2014; Vöhringer and Perlis 2016).

### Depression in overall morbidity

Of note, overall time in depressive phases of BD, and duration of depressive episodes are much greater than in mania or hypomania (“[hypo]mania”) (Kupka et al. 2007; De Dios et al. 2010). Moreover, morbidity has been surprisingly high in BD despite supposedly effective treatment. Indeed, BD patients averaged 45% of time ill during long-term follow-up, and depression accounted for 72% of time-ill, and somewhat more with BD-II (81%) than BD-I (70%) (Forte et al. 2015) (Table 1).

**Table 1:** Depressive morbidity in clinically treated bipolar disorder subjects. Data adapted from Forte et al. (2015), based on systematic review of studies involving adult patients treated by community standards

Measure	Bipolar I	Bipolar II	All bipolar
Studies	12	8	15
Subjects	2760	822	3582
Exposure (years)	7.78 [3.53–12.0]	8.28 [2.18–14.4]	7.89 [5.47–12.6]
%-Time depressed	30.6 [23.9–37.3]	35.9 [23.1–48.7]	31.8 [23.7–39.9]
Total %-time ill	43.7 [37.5–49.4]	43.2 [35.2–51.1]	43.6 [37.0–49.8]
%-of illness depressed	69.6 [60.4–78.9]	81.2 [71.3–91.0]	72.3 [62.9–81.7]

Data are means with 95% confidence intervals [CI]. Depression includes major episodes plus dysthymia

## Morbidity and disability

### Disability

Given the high proportion of time in depression among BD patients, depression is likely to be associated with dysfunction and disability, including limited academic achievement and decreased employment success. Perhaps 80% of BD patients experience some work-loss, and 30–40% experience prolonged unemployment during adult working years—much of that disability associated with depression (Zimmerman et al. 2010; Arvilommi et al. 2015).

### Co-occurring psychiatric disorders

Psychiatric conditions commonly encountered in BD patients include substance-abuse and anxiety disorders, as well as various personality disorders and temperament types (Goodwin and Jamison 2007; Pavlova et al. 2015; Preti et al. 2016; Messer et al. 2017; Stokes et al. 2017; Vázquez et al. 2017b; Post et al. 2018). Such concomitant conditions may meet standard diagnostic criteria, but whether they should be considered separate, “co-morbid” disorders vs. expressions of the range of psychopathology of BD remains unresolved (Yildiz et al. 2015; Vázquez et al. 2017b). Multiple diagnoses risk contributing to complexity and potential incoherence of treatment choices to compromise clinical care.

### General-medical morbidity and mortality

BD patients have increased risk of many general-medical disorders, including vascular conditions, with increased morbidity, disability and diminished longevity (McIntyre et al. 2007; Correll et al. 2017; Fornaro et al. 2017). In addition, obesity, diabetes, migraine, and some infectious diseases are more prevalent among BD patients (McIntyre et al. 2007; Almeida et al. 2018). With BD, risk of myocardial infarction was 37% greater (88% among women), stroke 60%, and congestive heart failure nearly 230% greater than in age-matched general populations (Wu et al. 2015; Fornaro et al. 2017; Tsai et al. 2017). Cardiovascular diseases are particularly frequent in association with BD disorder (Table 2) (Correll et al. 2017). Mediating factors include obesity, inactivity, diabetes or metabolic syndrome, and increased inflammatory factors—all with increased prevalence among BD patients (Vancampfort et al. 2013; Halaris 2017; Tsai et al. 2017), and at least in part attributable to treatments which may contribute to these risks (Baldessarini 2013; Correll et al. 2015).

**Table 2:** Risk of cardiovascular diseases in bipolar disorder patients vs. general population. Data adapted from Correll et al. (2017)

Outcome	Studies	Subjects	HR [95% CI]	*p*-value
Congestive heart failure	1	1397	2.27 [1.49–3.45]	< 0.0001
Cardiovascular mortality	3	179,651	1.65 [1.10–2.47]	0.02
Cerebrovascular disease	4	6,673,266	1.60 [0.99–2.57]	0.05
Any cardiovascular disease	10	7,058,912	1.57 [1.28–1.93]	< 0.0001
Coronary artery disease	4	6,808,812	1.16 [0.76–1.78]	0.49

Based on longitudinal studies with 8.4 [range: 1.8–30) years of follow-up. Hazard ratio (HR) is adjusted for six potential confounders; ranked by HR

With many general-medical disorders, BD patients have more adverse clinical outcomes and diminished life-expectancy, with all-cause mortality up to 15-times above general population rates, and rising (McIntyre et al. 2007; Ösby et al. 2018; Hällgren et al. 2018; Staudt-Hansen et al. 2019). Life-expectancy with BD is reduced by 12–15 years (Chesney et al. 2014). Factors associated with this decreased longevity include co-occurring substance abuse, smoking, and being overweight, unmarried, and having limited access to adequate medical care (Hjorthøj et al. 2015; Brietzke et al. 2017; Dickerson et al. 2018). The decreased longevity may be particularly associated with depression (Dickerson et al. 2018).

## Bipolar depression and suicide

### Suicidal risks

The reported international annual suicide rate averages 15.4/100,000 (0.015%/year), with wide regional variation (WHO 2018). The standardized mortality ratio (SMR) for suicide in BD is about 20 (Baldessarini et al. 2019a). By diagnosis, suicide risk ranks: bipolar disorders (BD-I = BD-II; especially with mixed or psychotic features) ≥ severe major depressive disorder with hospitalization > moderate depression among outpatients (Bachmann 2018; Hällgren et al. 2018; Baldessarini et al. 2019a). Risk for suicide and attempts is especially high in days following discharge from psychiatric hospitalization, in association with delay or lack of appropriate aftercare (Olfson et al. 2016; Large and Swaraj 2018; Forte et al. 2019).

In mood-disorder patients depressive-dysphoric phases are more associated with suicide than other illness states, especially if accompanied by mixed (hypomanic) features, co-occurring substance abuse, and following previous suicidal acts (Tondo et al. 1999, 2018; Baldessarini et al. 2019b). General population rates of suicide *attempts* average 0.2–0.6% per year, or approximately 36-times the suicide rate, and over 1%/year in BD (Kessler et al. 2005; Nock et al. 2008; Tondo et al. 2016; Baldessarini et al. 2019b). The ratio of suicide attempts/suicides (A/S), an *index of lethality* lower with more lethal intent or method, is only 5–10 in BD and MDD, or about five-times above lower than that for the general population (Tondo and Baldessarini 2015; Baldessarini et al. 2019b).

Among both BD-I and BD-II patients, especially with mixed or psychotic features, risk of suicidal behavior is among the highest of all psychiatric disorders despite supposedly effective treatments (Baldessarini et al. 2019b). This disparity almost certainly reflects great difficulty of treating depressive and mixed states in BD (Baldessarini et al. 2010c; Saunders and Hawton 2013; Forte et al. 2015). The remarkably prolonged delay of recognition and intervention in BD, sometimes for more than a decade, contrasts strikingly with observations that half of long-term risk of suicidal acts among BD patients occurred within the first 2–3 years of illness (Tondo and Baldessarini 2014, 2015).

### Suicide and treatment with antidepressants

Suicide cannot be “treated” but only prevented (Table 3). Research on treatments aimed at suicide prevention, not surprisingly, is very limited because of clinical and ethical problems arising if an inactive or ineffective treatment, such as placebo, were compared to an experimental intervention, with death as a potential outcome. In addition, it is virtually impossible to know when a suicide has been prevented, whereas suicidal acts or surrogate measures can be counted. Rarity of suicide, even among psychiatric patients, encourages research reliance on more prevalent measures related to suicide, including suicidal ideation, threats, self-injurious acts, or emergency interventions. However, the typically distant relationship of such measures to suicide limits their value in testing for therapeutic effects on suicide itself. Relating treatments to suicidal risks is further complicated by uncertain long-term adherence to recommended treatments (Isometsä 2005; Simon and Hales 2012; Baldessarini 2013; Ahmedani et al. 2014). Treatments for BD considered for possible suicide-prevention include antidepressants, anticonvulsants and lithium, antipsychotics, ECT, and psychosocial interventions (Table 3).

**Table 3:** Treatments aimed at reducing suicidal risk in bipolar disorder patients

Intervention	Timing	Findings	Comments
Antidepressants	Short-term benefits are not clear; long-term effects are virtually untested	Research findings are inconclusive. Suicidal risk may increase with agitation, and in youth but may be lower in older adults	Studies lack long-term randomization with suicidal acts as an explicit outcome measure
Antipsychotics	Short-term benefits are not adequately tested. Clozapine is probably beneficial long-term in schizophrenia (with FDA approval) but untested in BD	Except for clozapine, testing remains inadequate and inconclusive	Effects of clozapine rely mainly on a single randomized trial vs. olanzapine, without reduction of mortality
Anticonvulsants	Short-term effects are not established; long-term benefits have been proposed	Valproate most studied; anticonvulsants may be less effective than lithium	Studies lack suicidal acts as an explicit outcome
Lithium	Very likely effective long-term	Consistent decrease of risk of suicide and attempts in controlled and uncontrolled studies; not clear if effect is via reducing risk of depression, impulsivity, or specific anti-suicidal action	Even randomized trials lack suicidal behavior as explicit outcome measure. Long-term acceptance and tolerance suggests some self-selection; potentially toxic on overdose
Other pharmacological treatments	Only short-term effects have been tested	Ketamine can reduce suicidal ideation; effects on suicidal behavior are untested; newer NMDA agents untested	Ketamine has a short-term antidepresant effect in BD
Other somatic treatments	If there are benefits, they are probably short-term	ECT, magnetic, vagal nerve, or deep-brain stimulations can benefit depression	Inadequate testing vs. suicidal behavior specifically
Psychotherapies	Effects not established, but widely assumed to be helpful clinically	Cognitive-behavioral, dialectic and interpersonal methods best studied, but research results for suicide are inconclusive	Psychotherapy involves self-selection

References to relevant studies are provided in the text

Strong association of suicidal behavior or acts with depression suggests that treatment with antidepressants might reduce suicidal risk, though most studies have yielded inconsistent evidence. Most were not designed to test for suicidal behavior as an explicit outcome measure rather than as an incidental and passively reported “adverse event” (Möller 2006; Tondo and Baldessarini 2015; Baldessarini et al. 2020). Too, some patients can *worsen* clinically when given an antidepressant, and the treated depressive episode can be accompanied by agitation, dysphoria, restlessness, irritability, anger, insomnia, behavioral disinhibition, or other mixed features, with increased risk of suicidal behavior (Tondo et al. 1999, 2018; Maj et al. 2006; Simon and Hales 2012; Pacchiarotti et al. 2013). In addition, abrupt or rapid discontinuation of antidepressant treatment markedly increases early risk of new depression, and might increase suicidal risk (Baldessarini et al. 2010b).

Several studies have found only minor associations of antidepressant treatment and suicidal behaviors, mainly with MDD (Beasley et al. 1991; Acharya et al. 2006; Möller 2006; Tondo et al. 2008; Khan et al. 2011; Tondo and Baldessarini 2015; Braun et al. 2016). Other findings noted increased risk of suicidal acts in juveniles and young adults but decreased risk in older adults (Hammad et al. 2006; Laughren et al. 2006; Bridge et al. 2007; Barbui et al. 2009; Saunders and Hawton 2013; Braun et al. 2016). However, most such studies lacked explicit, validated, predefined outcome measures pertinent to suicide.

In our experience, emergence of new suicidal behaviors among mood-disordered adults treated with sustained antidepressant treatment in clinical settings was infrequent, involving perhaps 5/1000 patients/year (Tondo et al. 2008). Nevertheless, risks of clinical worsening with antidepressants, as well as the possibility that acute depression may be the initial episode of BD, should be considered and monitored at any age, especially early in antidepressant treatment.

### Lithium treatment and suicide

An association of reduced risk of suicides and attempts during long-term treatment with lithium in BD is supported consistently by most (Müller-Oerlinghausen et al. 2006; Baldessarini and Tondo 2008; Tondo and Baldessarini 2014, 2015, 2018; Roberts et al. 2017; Smith and Cipriani 2017; Felber et al. 2018), but not all studies (Marangell et al. 2008; Oquendo et al. 2011). At least 10 placebo-controlled, randomized trials not specifically designed with suicide risk as the primary outcome measure, but involving more than 110,000 person-years of risk, found five- to sixfold reductions in suicidal acts (Tondo et al. 1998, 2001; Angst et al. 2005; Cipriani et al. 2005; Baldessarini et al. 2006; Lauterbach et al. 2008; Khan et al. 2011). Based on such studies, several expert reports recommend long-term lithium treatment to limit risk of suicidal behavior in BD patients (Wasserman et al. 2012; Lewitzka et al. 2013; Yatham et al. 2018).

### Anticonvulsants and suicide

Few studies directly compare suicidal risks during treatment with alternatives to lithium, including anticonvulsants, and findings are largely inconsistent and inconclusive (Thies-Flechtner et al. 1996; Goodwin et al. 2003; Yerevanian et al. 2007; Baldessarini and Tondo 2009; Chen et al. 2019). The FDA (2008) proposed that some anticonvulsants may even be associated with increased risk of suicidal behavior, at least in epilepsy patients, though probably not in psychiatric applications (Yerevanian et al. 2007; Gibbons et al. 2009). Meta-analysis of suicidal behavior with lithium vs. several anticonvulsants (mainly valproate) in six direct comparisons involving over 30,000 patients found nearly three-fold greater reductions with lithium (Baldessarini and Tondo 2009).

### Antipsychotics and suicide

Antipsychotic drugs remain little-evaluated for effects on suicidal behavior (Simon and Hales 2012). However, one study found no difference in relatively short-term risk of suicides or attempts during treatment of > 10,000 psychotic patients with either first- or second-generation antipsychotics (FGAs or SGAs) vs. placebo (Khan et al. 2001). In addition, mortality risk was not increased in nearly 109,000 schizophrenia subjects given antipsychotic drugs (Schneider-Thoma et al. 2018), but was greater without antipsychotic treatment in another study of over 2200 such patients (Tiihonen et al. 2006). The InterSePT study comparing suicide-related behavior in schizophrenia patients at high risk for suicide provided strong support for an antisuicidal effect of clozapine compared to olanzapine (Meltzer et al. 2003). Clozapine has not been evaluated adequately in the treatment of BD patients, although it may have antimanic or mood-stabilizing effects (Li et al. 2015). Studies of SGAs with antidepressant effects in BD patients, in particular, require assessment for effects on suicide.

### Other treatments and suicide

Evidence is growing that the glutamate NMDA-receptor antagonist ketamine and its active *S*-enantiomer (esketamine) can exert rapid, short-term reduction of suicidal ideation along with rapid reduction of symptoms of depression, including in BD patients, although effects on suicidal behavior are uncertain (Parsaik et al. 2015; Grunebaum et al. 2017; Wilkinson et al. 2018). There is considerable uncertainty about how to continue use of racemic or *S*-ketamine following initial benefits, and some concern that its discontinuation may provoke adverse clinical responses (Schatzberg 2019). ECT often appears to be lifesaving in suicidal emergencies but lacks evidence of sustained antisuicidal efficacy (Fink et al. 2014). Other methods of external electrical or magnetic stimulation of brain, vagal nerve stimulation, and deep brain stimulation are being investigated or introduced for the treatment of otherwise treatment-resistant depression but remain to be tested adequately for specific effects on suicidal behavior, particularly in BD.

Additional interventions of potential value include emergency hospitalization (Zalsman et al. 2016) as well as psychotherapies, in particular cognitive-behavioral, dialectic, and interpersonal methods, which can improve depressive symptoms and may reduce suicidal risk (Brown et al. 2005; Zalsman et al. 2016; McCauley et al. 2018; Baldessarini et al. 2020). However, results from studies of psychosocial interventions may be limited by the self-selection of patients who adhere to such prolonged treatments.

## Treatment of bipolar depression

As noted, depressive, dysthymic, and mixed states account for the majority of illness-burden in BD, and are strongly predicted by initial depressive, mixed, or anxious episodes (Goodwin and Jamison 2007; Yildiz et al. 2015; Forte et al. 2015; Baldessarini et al. 2014, 2019a). Remarkably few treatments are proved to be highly and consistently effective in acute episodes of bipolar depression, and there is even less evidence supporting substantial long-term protection from recurrences (Table 4). In particular, there is continued controversy about the value and risks of antidepressant drugs in bipolar depression (Pacchiarotti et al. 2013; McGirr et al. 2016). Lack of highly effective treatments encourages widespread drug-combinations and other off-label treatments largely untested for effectiveness and safety.

**Table 4:** Placebo-controlled trials for acute depression in bipolar disorder

Treatments	Subjects (n)	Responders/subjects (%)		RR [95%CI]
		Drug	Placebo	
Anticonvulsants [10 trials, 3 agents]	1281	313/657 [47.6%]	181/624 [29.0%]	1.61 [1.39–1.87]
Antidepressants [12 trials, 11 agents]	1895	383/803 [48.9%]	419/1092 [38.4%]	1.32 [1.07–1.62]
Antipsychotics [13 trials, 6 agents]	6044	2135/3859 [55.3%]	904/2185 [41.4%]	1.28 [1.09–1.51]
Lithium [1 trial, 1 agent]	265	85/136 [62.5%]	72/129 [55.8%]	1.12 [0.92–1.37]
Pooled/totals [36 trials, 21 agents]	9485	2926/5455 [54.4%]	1576/4030 [39.4%]	1.37 [1.30–1.44]

Dropout rates (average: 32.9% [28.0–37.8]) were similar across treatments and with drug or placebo. Response typically involved ≥ 50% improvement in depression symptom ratings. By separate random-effects meta-analysis, antidepressants were statistically more effective than placebo (RR = 1.32 [1.07–1.87]; *z *= 2.65, *p *= 0.008), as were the other agents (RR = 1.34 [1.17–1.53]; *z *= 431, *p *< 0.0001). The overall weighted average drug vs. placebo difference (RR = 1.37) was highly significant (χ^2^ = 196, *p *< 0.0001). Antidepressant dose averaged 172 [146–198] mg/day imipramine-equivalent (Baldessarini 2013). Antidepressant monotherapy trials yielded greater drug/placebo differences than with addition to a mood-stabilizer (RR = 1.64 [1.05–2.56] vs. 1.18 [0.96–1.46]). Of note, in 23/36 trials (63.9%) drug was *not* statistically superior to placebo. Results are ranked by drug/placebo Risk Ratios (RR). [References: Nemeroff et al. (2001); Tohen et al. (2003); Shelton and Stahl (2004); Agosti and Stewart (2008); McElroy et al. (2010); McGirr et al. (2016); Yatham et al. (2018); Vázquez et al. (2017a); Baldessarini et al. (2019b)]

Relative paucity of experimental treatment studies for bipolar depression may reflect a broadly accepted view that “major depression” is similar in its clinical characteristics as well as treatment responses in BD and MDD (Baldessarini 2013). Instead, their characteristics differ, e.g., in family history, sex-distribution, onset-age, long-term diagnostic stability, episode duration, recurrence rates, and treatment-responses (Baldessarini et al. 2010a, c). The assumption of similarity probably contributes to the rarity of direct comparisons of treatment responses with depression in BD vs. MDD, and leaves bipolar depression as a leading challenge for psychiatric therapeutics (Goodwin et al. 2016; Baldessarini et al. 2019b, 2020).

### Antidepressants for bipolar depression

Ease and relative safety of treating depressive episodes with modern antidepressants, and strenuous efforts to minimize or avoid depression by BD patients and clinicians, have made antidepressants the leading treatment provided to BD patients (Baldessarini et al. 2008, 2019b). Nevertheless, there is a striking paucity of therapeutic experimentation and inconsistent findings, despite more than a half-century of use of antidepressant drugs to treat “depression,” with particularly serious gaps regarding dysthymia and dysphoria, mixed features, and long-term prophylaxis for bipolar depression (Ghaemi et al. 2008, 2010; Sidor and MacQueen 2012; Baldessarini 2013; Pacchiarotti et al. 2013; Fountoulakis et al. 2017; Liu et al. 2017a; Gitlin 2018). Many experts advise caution in using antidepressants, particularly for BD-I patients to avoid potentially dangerous mood-switches, and encourage their use, if necessary, only with mood-stabilizing agents or SGAs, and without current mixed features or agitation (Pacchiarotti et al. 2013; Tondo et al. 2013; Goodwin et al. 2016; Yatham et al. 2018).

Well-designed, controlled, monotherapy trials of antidepressants for acute bipolar depression are surprisingly few, vary in size and quality, and yield inconsistent findings (Table 4) (Vázquez et al. 2011; Tondo et al. 2013; Gitlin 2018; Yatham et al. 2018). Two large trials found no additional improvement in bipolar depression by adding paroxetine or bupropion to mood-stabilizing or antipsychotic drugs (Sachs et al. 2007; McElroy et al. 2010). Two meta-analyses including these and the few other relevant trials supported possible efficacy of various antidepressants in bipolar depression (Gijsman et al. 2004; Vázquez et al. 2013); another did not (Sidor and MacQueen 2012). Several direct comparisons found similar antidepressant responses in depressed BD and MDD patients (Vázquez et al. 2011). Another comparison of clinical responses in large samples of depressed BD-I, BD-II, or MDD patients also found only minor differences in response or remission and low risk of mood-switching in these disorders, provided that subjects with agitation or even minor mixed features were excluded (Tondo et al. 2013).

Impressions that antidepressants may be less effective in acute bipolar depression than in MDD may, to some extent, reflect adverse effects of treatment, including worsening of agitation, anger, or dysphoria, interpreted as failure of depression to respond (Tondo et al. 2013). Our findings from available randomized, controlled trials support the impression that antidepressant treatment has yielded a significant, 32% superiority over placebo for acute bipolar depression, with moderately high heterogeneity of outcomes (Table 4). Despite this limited and inconsistent body of research, it is evidently widely assumed clinically that antidepressants may be appropriate for some BD patients, and especially safe for BD-II depression (Baldessarini et al. 2008; Amsterdam and Shults 2010; Undurraga et al. 2012; Altshuler et al. 2017; Gitlin 2018). Selection of BD candidates for clinical antidepressant treatment may usefully be guided by previous beneficial and tolerated responses, relatively less severe or nonrapidly cycling illness, relatively few previous depressions, lack of switching from depression to mania, or of current agitation or even minor mixed features (Pacchiarotti et al. 2013; Tondo et al. 2013; Baldessarini et al. 2019b). Research on biomarkers associated with response to antidepressants is ongoing and may help in identifying more effective treatments for various types of depression (Gadad et al. 2018).

### Antidepressants and mood switching

There is widespread concern that antidepressant treatment for bipolar depression risks switching into potentially dangerous agitation or mania, especially in BD-I (Bond et al. 2008; Undurraga et al. 2012). Such risk is more associated with the long-term BD course-pattern of depression followed by mania before a stable interval (“DMI”) than the opposite (“MDI”) (Koukopoulos et al. 2013). However, it is difficult to distinguish spontaneous from antidepressant-associated switching in BD, mean rates of which are similar (13.8% [12.2–15.3] vs. 15.3% [14.5–16.1]) (Tondo et al. 2010). Though it is plausible to expect mood-stabilizing and antipsychotic drugs to prevent mood-switching with antidepressants, required randomized comparisons are lacking (Tondo et al. 2010; Baldessarini et al. 2019b). Trials of antidepressants have found little difference in risk of new mania between antidepressants and placebo, with or without a mood-stabilizer included, although exposure times were short (Liu et al. 2017a). However, one study found that switching in BD was 2.8-times greater within 9 months after adding an antidepressant, but not if a mood-stabilizer also was used (Viktorin et al. 2014), and switching risk was increased in the rare long-term trials with an antidepressant included in treatment (Ghaemi et al. 2008).

An evident clinical consensus is that antidepressants be used for BD only cautiously, with short-acting agents given in moderate, slowly increased doses, briefly, and with effective mood-stabilizing co-treatment, while monitoring for emerging hypomania. It seems prudent that antidepressants, especially tricyclics and some SNRIs, be used very cautiously for bipolar depression, especially in BD-I patients, and perhaps avoided altogether with a history of mood-switching during antidepressant treatment, rapid-cycling without antidepressant treatment, or if mixed symptoms are present (Tondo et al. 2010; Pacchiarotti et al. 2013).

### Mood-stabilizers

Several anticonvulsants have been used widely for BD, based on secure evidence of short-term antimanic effects (carbamazepine and valproate) or long-term reduction of risk of depressive recurrences (lamotrigine) (Baldessarini 2013; Geddes and Miklowitz 2013; Reinares et al. 2013). Such treatment choices are encouraged by seeming simpler than treatment with lithium (Baldessarini 2013; Vázquez et al. 2014). For divalproex monotherapy, 4 small trials suggest possible value in acute bipolar depression (Table 4), but it remains FDA-unapproved for depression or long-term treatment in BD. Evidence that lamotrigine is effective in acute bipolar depression rests on pooling inconsistent data, including from individually failed trials vs. placebo (Table 4) (Solmi et al. 2016). Lamotrigine is FDA-approved only for long-term prophylaxis in BD, with partial effectiveness against recurrences of depression but little efficacy against acute or recurrent mania (Frye et al. 2011; Baldessarini 2013). Moreover, slow dose-increases to avoid potentially serious dermatological reactions limit practicality of off-label use of lamotrigine in acute bipolar depression. Evidence concerning carbamazepine for short- or long-term use for bipolar depression is very limited (Table 4), and controlled trials for other anticonvulsants in BD are lacking (Reinares et al. 2013; Selle et al. 2014).

Despite use of lithium as a fundamental treatment for BD for more than six decades, and its position as a first-line treatment in some expert guidelines (Goodwin et al. 2016; Yatham et al. 2018), it remains virtually untested for acute bipolar depression. Lithium was included as a third-arm of a trial in acute bipolar depression designed primarily to test quetiapine, with little benefit (Table 4) (Young et al. 2010). Nevertheless, lithium has some long-term effectiveness against recurrences of bipolar depression as well as greater prophylactic effects against [hypo]mania (Baldessarini 2013; Bschor 2014; Yatham et al. 2018), and benefits in mixed episodes in BD (Sani and Fiorillo 2019). Moreover, as noted, lithium may reduce risk of suicide substantially in BD patients (Tondo et al. 2001; Baldessarini et al. 2006; Cipriani et al. 2013; Tondo and Baldessarini 2014, 2018; Song et al. 2017).

### Second-generation antipsychotics

SGAs, including cariprazine, lurasidone, olanzapine-fluoxetine, and quetiapine are currently the only FDA-approved medicines for short-term treatment of acute depressive episodes in BD (Baldessarini 2013; Selle et al. 2014; Earley et al. 2019; Ragguett and McIntyre 2019). Of these, only quetiapine has outperformed placebo consistently in several trials, with similar results for doses of 300 vs. 600 mg/day, and only the lower dose is FDA-approved (McElroy et al. 2010). Olanzapine-fluoxetine was superior to placebo, whereas olanzapine alone was less effective (Tohen et al. 2003). Unsurprisingly, as both olanzapine and quetiapine are antimanic, they have yielded somewhat *lower* risks of mood-switching than placebo (Selle et al. 2014). Most of these responses in acute bipolar depression have been modest (Table 4), and possible long-term protective effects require further study. Of note, beneficial effects in bipolar depression are not a class-effect of all SGAs (Taylor et al. 2014). In effective doses, antipsychotics risk adverse effects that include excessive sedation as well as distressing restlessness (akathisia) (Brown et al. 2006; Tamayo et al. 2010). Although risks of tardive dyskinesia with most SGAs are far lower than with FGAs (Tarsy et al. 2010; Carbon et al. 2017), their greatly increasing use and broadening indications may risk increased numbers of cases of even this uncommon adverse outcome (Pompili et al. 2016). Moreover, risks of weight-gain, type-2 diabetes, and other features of metabolic syndrome (hyperlipidemia, hypertension) are encountered with some SGAs (particularly olanzapine and quetiapine), sometimes rapidly (Centorrino et al. 2012; Baldessarini 2013; Vázquez et al. 2015). These medically important adverse effects tend to limit the potential value of SGAs for prophylactic treatment against recurrences of bipolar depression (Vázquez et al. 2014, 2015; Fountoulakis et al. 2017). In summary, cariprazine, lurasidone, and quetiapine, as well as olanzapine-fluoxetine are effective in acute bipolar depression, though with some risks, and they need further testing for long-term, prophylactic effects against bipolar depression.

### Other treatments

Growing numbers of novel pharmacological treatments for depression are under investigation; some may be of value in BD, including drugs that act at synaptic transmission systems mediated by amino acid neurotransmitters glutamate and GABA. They include the NMDA-glutamate receptor antagonist ketamine and newer pharmacologically similar agents (e.g., apimostinel, rapastinel) (Dhir 2017; Garay et al. 2017; Grady et al. 2017; Grunebaum et al. 2017; Ragguett et al. 2019; Wilkinson and Sanacora 2019). Given apparent association of postpartum mood disorders and BD (Liu et al. 2017b), neurosteroids that interact with GABA_A_ receptors and found effective for postpartum depression (e.g., brexanolone) may be of interest for bipolar depression (Martinez-Botella et al. 2017; Scott 2019). Agents of less certain value include polyunsaturated fatty acids, anti-inflammatory agents, and probiotics (Vázquez et al. 2017a).

Among nonpharmacological treatments, acute bipolar depression is responsive to ECT (Itagaki et al. 2017; Perugi et al. 2017; Bahji et al. 2019), although optimal treatment to follow successful ECT remains uncertain. Other biomedical treatments may be of value in bipolar depression. Intense light therapy and sleep deprivation are plausible candidates that require adequate testing in BD (Tseng et al. 2016; Suzuki et al. 2018). Vagal nerve stimulation (VNS) is FDA-approved for treatment-resistant depression, with evidence of efficacy in depression of BD and MDD (Cimpianu et al. 2017; Conway et al. 2018), though with some risk of inducing mania (Salloum et al. 2017). Repeated transcranial magnetic stimulation (rTMS) and various forms of electrical stimulation of brain from the surface or through stereotaxically placed deep-brain electrodes remain experimental for bipolar depression (Nierenberg et al. 2008; Vázquez et al. 2017a; Widge et al. 2018; Filkowski and Sheth 2019).

Finally, several manual-based, replicable forms of psychotherapy, alone or added to antidepressants, have shown promise for treating BD patients (Bouwkamp et al. 2013; McMahon et al. 2016; Salcedo et al. 2016; Lovas and Schuman-Olivier 2018; Yatham et al. 2018).

## Conclusions

Depression, dysthymia, and dysphoria in BD represent major, only partially solved, clinical challenges (Table 5). As the main unresolved illness in treated BD, bipolar depression is associated with excess morbidity as well as mortality from co-occurring general-medical disorders and very high suicide risk. Suicide risk in BD exceeds general-population rates by 20-fold and is strongly associated with depressive phases, especially with mixed or psychotic features. Treatments proposed to reduce suicide risk notably include lithium. Treatment of bipolar depression is far less well investigated than MDD, and the value and tolerability of standard antidepressants for bipolar depression remain controversial. Evidence of efficacy in bipolar depression of mood-stabilizing agents, including lithium and several anticonvulsants (except lamotrigine, long-term) remains far less substantial than for several SGAs. All available pharmacological treatments used for bipolar depression have limited efficacy and risk adverse metabolic or neurological effects. Overall, we strongly encourage renewed efforts to consider bipolar depression as distinct from depression in MDD and to seek more effective treatments especially for long-term prophylaxis aimed at reducing morbidity and mortality.

**Table 5:** Current status of depression in bipolar disorder

Depression in bipolar disorder (BD) is the major residual psychiatric morbidity with available treatments, accounting for three-quarters of the 40–50% long-term time-ill
Unresolved morbidity, and especially depression, is associated with excess medical morbidity, including metabolic syndrome and cardiovascular disease, with increased mortality
Suicide risk in BD is similar in types I and II BD, greater than in most other psychiatric disorders, ca. 20-times above general population rates, and strongly associated with depression, especially with agitation (mixed-dysphoric states), and in the days–weeks following hospital discharge
Predicting suicide in BD clinically is limited regarding individuals and timing
Treatments proposed to prevent suicidal behavior in BD include lithium, clozapine, and possibly ketamine and psychotherapies, which all require further study
Therapeutics of bipolar depression is far less well developed than for nonbipolar major depression, probably reflecting lack of recognition of differences between bipolar and unipolar depression
The short-term value and safety of antidepressant treatment for bipolar depression remains controversial, and long-term value remains virtually untested; it is best avoided with ongoing dysphoric agitation or mixed features
Some modern antipsychotics are effective in bipolar depression short-term; lithium and lamotrigine have modest prophylactic value long-term but are not adequately tested short-term; other anticonvulsant mood-stabilizers have very limited evidence of short- or long-term efficacy in bipolar depression
All available treatments for bipolar depression have risks of adverse metabolic or neurological effects; valproate and carbamazepine are also highly teratogenic

## Data Availability

Relevant data are provided in the tables within the report and in the references cited.

## References

[CR1] Acharya N, Rosen AS, Polzer JP (2006). Duloxetine: meta-analyses of suicidal behaviors and ideation in clinical trials for major depressive disorder. J Clin Psychopharmacol.

[CR2] Agosti V, Stewart JW (2008). Hypomania with and without dysphoria: comparison of comorbidity and clinical characteristics of respondents from a national community sample. J Affect Disord.

[CR3] Ahmedani BK, Simon GE, Stewart C (2014). Healthcare contacts in the year before suicide death. J Gen Int Med.

[CR4] Almeida OP, Hankey GJ, Yeap BB, Golledge J, Flicker L (2018). Older men with bipolar disorder: clinical associations with early and late illness. Int J Geriatr Psychiatry.

[CR5] Altshuler LL, Sugar CA, McElroy SL (2017). Switch rates during acute treatment for bipolar II depression with lithium, sertraline, or the two combined: randomized, double-blind comparison. Am J Psychiatry.

[CR6] Amsterdam JD, Shults J (2010). Efficacy and safety of long-term fluoxetine vs. lithium monotherapy of bipolar II disorder: randomized, double-blind, placebo-substitution study. Am J Psychiatry.

[CR7] Angst J, Angst F, Gerber-Werder R, Gamma A (2005). Suicide in 406 mood-disorder patients with and without long-term medication: a 40 to 44 years’ follow-up. Arch Suicide Res.

[CR8] Arvilommi P, Suominen K, Mantere O, Valtonen H, Leppamaki S, Isometsa E (2015). Predictors of long-term work disability among patients with type I and II bipolar disorder: prospective 18-month follow-up study. Bipolar Disord.

[CR9] Bachmann S (2018). Epidemiology of suicide and the psychiatric perspective. Int J Environ Res Public Health.

[CR10] Bahji A, Hawken ER, Sepehry AA, Cabrera CA, Vázquez GH (2019). ECT beyond unipolar major depression: systematic review and meta-analysis of electroconvulsive therapy in bipolar depression. Acta Psychiatr Scand.

[CR11] Baldessarini RJ (2013). Chemotherapy in psychiatry.

[CR12] Baldessarini RJ, Tondo L (2008). Lithium and suicidal risk. Bipolar Disord.

[CR13] Baldessarini RJ, Tondo L (2009). Suicidal risks during treatment of bipolar disorder patients with lithium versus anticonvulsants. Pharmacopsychiatry.

[CR14] Baldessarini RJ, Tondo L, Davis P (2006). Decreased risk of suicides and attempts during long-term lithium treatment: meta-analytic review. Bipolar Disord.

[CR15] Baldessarini RJ, Henk H, Sklar A, Chang J, Leahy L (2008). Psychotropic medications for patients with bipolar disorder in the United States: polytherapy and adherence. Psychiatr Serv.

[CR16] Baldessarini RJ, Salvatore P, Khalsa HM (2010). Morbidity in 303 first-episode bipolar I disorder patients. Bipolar Disord.

[CR17] Baldessarini RJ, Tondo L, Ghiani C, Lepri B (2010). Illness risk following rapid vs. gradual discontinuation of antidepressants. Am J Psychiatry.

[CR18] Baldessarini RJ, Vieta E, Calabrese JR, Tohen M, Bowden C (2010). Bipolar depression: overview and commentary. Harv Rev Psychiatry.

[CR19] Baldessarini RJ, Undurraga J, Vázquez GH (2012). Predominant recurrence polarity among 928 adult international bipolar I disorder patients. Acta Psychiatr Scand.

[CR20] Baldessarini RJ, Faedda GL, Offidani E (2013). Antidepressant-associated mood-switching and transition from unipolar major depression to bipolar disorder. J Affect Disord.

[CR21] Baldessarini RJ, Tondo L, Visioli C (2014). First-episode types in bipolar disorder: predictive associations with later illness. Acta Psychiatr Scand.

[CR22] Baldessarini RJ, Pérez J, Salvatore P, Trede K, Maggini C, Yildiz A, Nemeroff C, Ruiz P (2015). Chapt 1: History of bipolar manic-depressive disorder. The bipolar book: history, neurobiology, and treatment.

[CR23] Baldessarini RJ, Tondo L, Pinna N, Nuñez GH, Vázquez GH (2019). Suicidal risk factors in major affective disorders. Br J Psychiatry.

[CR24] Baldessarini RJ, Tondo L, Vázquez GH (2019). Pharmacological treatment of adult bipolar disorder. Mol Psychiatry.

[CR25] Baldessarini RJ, Tondo L, Vázquez GH, Pompili M, McIntyre RS, Fiorillo A, Sartorius N (2020). Chapt 4: Unmet needs in psychiatry: bipolar depression. New directions in psychiatry.

[CR26] Barbui C, Esposito E, Cipriani A (2009). Selective serotonin reuptake inhibitors and risk of suicide: systematic review of observational studies. CMAJ.

[CR27] Barbuti M, Pacchiarotti I, Vieta E (2017). Antidepressant-induced hypomania/mania in patients with major depression: evidence from the BRIDGE-II-MIX study. J Affect Disord.

[CR28] Beasley CM, Dornseif BE, Bosomworth JC (1991). Fluoxetine and suicide: meta-analysis of controlled trials of treatment for depression. BMJ.

[CR29] Bond DJ, Noronha M, Kauer-Sant’Anna M, Lam RW, Yatham LN (2008). Antidepressant-associated mood elevations in bipolar II disorder compared with bipolar I disorder and major depressive disorder: systematic review and meta-analysis. J Clin Psychiatry.

[CR30] Bouwkamp CG, de Kruiff ME, van Troost TM (2013). Interpersonal and social rhythm group therapy for patients with bipolar disorder. Int J Group Psychother.

[CR31] Braun C, Bschor T, Franklin J, Baethge C (2016). Suicides and suicide attempts during long-term treatment with antidepressants: meta-analysis of 29 placebo-controlled studies including 6934 patients with major depressive disorder. Psychother Psychosom.

[CR32] Bridge JA, Iyengar S, Salary CB (2007). Clinical response and risk for reported suicidal ideation and suicide attempts in pediatric antidepressant treatment: meta-analysis of randomized controlled trials. JAMA.

[CR33] Brietzke E, Mansur RB, McIntyre RS (2017). Impact of inequalities in healthcare on the mortality risk of individuals with severe mental illnesses. Braz J Psychiatry.

[CR34] Brown GK, Ten Have T, Henriques GR, Xie SX, Hollander JE, Beck AT (2005). Cognitive therapy for the prevention of suicide attempts: randomized controlled trial. JAMA.

[CR35] Brown EB, McElroy SL, Keck PE (2006). Seven-week, randomized, double-blind trial of olanzapine/fluoxetine combination versus lamotrigine in the treatment of bipolar I depression. J Clin Psychiatry.

[CR36] Bschor T (2014). Lithium in the treatment of major depressive disorder. Drugs.

[CR37] Bschor T, Angst J, Azorin JM (2012). Are bipolar disorders underdiagnosed in patients with depressive episodes? Results of the multicenter BRIDGE screening study in Germany. J Affect Disord.

[CR38] Carbon M, Hsieh C-H, Kane JM, Correll CU (2017). Tardive dyskinesia prevalence in the period of second-generation antipsychotic use, meta-analysis. J Clin Psychiatry.

[CR39] Centorrino F, Masters GA, Talamo A, Baldessarini RJ, Öngür D (2012). Metabolic syndrome in psychiatrically hospitalized patients treated with antipsychotics and other psychotropics. Hum Psychopharmacol.

[CR40] Chen TY, Kamali M, Chu CS, Yeh CB, Huang M, Mao WC, Lin Y, Chen YW, Tseng PT, Hsu CY (2019). Divalproex and its effect on suicide risk in bipolar disorder: systematic review and meta-analysis of multinational observational studies. J Affect Disord.

[CR41] Chesney E, Goodwin GM, Fazel S (2014). Risks of all-cause and suicide mortality in mental disorders: meta-review. World Psychiatry.

[CR42] Cimpianu CL, Strube W, Falkai P, Palm U, Hasan A (2017). Vagus nerve stimulation in psychiatry: systematic review of the available evidence. J Neural Transm.

[CR43] Cipriani A, Pretty H, Hawton K, Geddes JR (2005). Lithium in the prevention of suicidal behavior and all-cause mortality in patients with mood disorders: systematic review of randomized trials. Am J Psychiatry.

[CR44] Cipriani A, Hawton K, Stockton S, Geddes JR (2013). Lithium in the prevention of suicide in mood disorders: updated systematic review and meta-analysis. BMJ Clin Res.

[CR45] Conway CR, Kumar A, Xiong W, Bunker M, Aaronson ST, Rush AJ (2018). Chronic vagus nerve stimulation significantly improves quality of life in treatment-resistant major depression. J Clin Psychiatry.

[CR46] Correll CU, Detraux J, De Lepeleire J, De Hert M (2015). Effects of antipsychotics, antidepressants and mood stabilizers on risk for physical diseases in people with schizophrenia, depression, or bipolar disorder. World Psychiatry.

[CR47] Correll CU, Solmi M, Veronese N (2017). Prevalence, incidence and mortality from cardiovascular disease in patients with pooled and specific severe mental illness: a large-scale, meta-analysis of 3,211,768 patients and 113,383,368 controls. World Psychiatry.

[CR48] Cuellar AK, Johnson SL, Winters R (2005). Distinctions between bipolar and unipolar depression. Clin Psychol Rev.

[CR49] De Dios C, Ezquiaga E, Garcia A, Soler B, Vieta E (2010). Time spent with symptoms in a cohort of bipolar disorder outpatients in Spain: prospective, 18-month follow-up study. J Affect Disord.

[CR50] Dhir A (2017). Investigational drugs for treating major depressive disorder. Expert Opin Investig Drugs.

[CR51] Dickerson F, Origoni A, Schroeder J (2018). Natural cause mortality in persons with serious mental illness. Acta Psychiatr Scand.

[CR52] Drancourt N, Etain B, Lajnef M, Henry C, Raust A, Cochet B, Mathieu F, Gard S, Mbailara K, Zanouy L, Kahn JP, Cohen RF, Wajsbrot-Elgrabli O, Leboyer M, Scott J, Bellivier F (2013). Duration of untreated bipolar disorder: missed opportunities on the long road to optimal treatment. Acta Psychiatr Scand.

[CR53] Earley W, Burgess MV, Rekeda L (2019). Cariprazine treatment of bipolar depression: randomized double-blind placebo-controlled, phase-3 study. Am J Psychiatry.

[CR54] FDA (US Food and Drug Administration). Antiepileptic drugs and suicidality 2008. Anticon, https://www.fda.gov/downloads/drugs/drugsafety/postmarketdrugsafetyinformationforpatientsandproviders/ucm192556.pdf. Accessed 22 Dec 2018.

[CR55] Felber W, Bauer M, Lewitzka U, Müller-Oerlinghausen B (2018). Lithium clinics in Berlin and Dresden: 50-year experience. Pharmacopsychiatry.

[CR56] Filkowski MM, Sheth SA (2019). Deep brain stimulation for depression: emerging indication. Neurosurg Clin N Am.

[CR57] Fink M, Kellner CH, McCall WV (2014). Role of ECT in suicide prevention. J ECT.

[CR58] Fornaro M, Solmi M, Veronese N (2017). Burden of mood-disorder/cerebrovascular disease comorbidity: essential neurobiology, psychopharmacology, and physical activity interventions. Int Rev Psychiatry.

[CR59] Forte A, Baldessarini RJ, Tondo L, Vázquez G, Pompili M, Girardi P (2015). Long-term morbidity in bipolar-I, bipolar-II, and major depressive disorders. J Affect Disord.

[CR60] Forte A, Buscaioni A, Fiorillo A, Pompili M, Baldessarini RJ (2019). Suicidal risk following hospital discharge: review. Harv Rev Psychiatry.

[CR61] Fountoulakis KN, Vieta E, Young A (2017). Unmet needs in the treatment of bipolar disorder and recommendations for future research. Int J Neuropsychopharmacol.

[CR62] Frye MA, Ha K, Kanba S (2011). International consensus group on depression prevention in bipolar disorder. J Clin Psychiatry.

[CR63] Gadad BS, Jha MK, Czysz A (2018). Peripheral biomarkers of major depression and antidepressant treatment response: current knowledge and future outlooks. J Affect Disord.

[CR64] Garay RP, Zarate CA, Charpeaud T (2017). Investigational drugs in recent clinical trials for treatment-resistant depression. Expert Rev Neurother.

[CR65] Geddes JR, Miklowitz DJ (2013). Treatment of bipolar disorder. Lancet.

[CR66] Ghaemi SN, Wingo AF, Filkowski MA, Baldessarini RJ (2008). Long-term antidepressant treatment in bipolar disorder: meta-analyses of benefits and risks. Acta Psychiatr Scand.

[CR67] Ghaemi SN, Ostacher MM, El-Mallakh RS (2010). Antidepressant discontinuation in bipolar depression: randomized clinical trial of long-term effectiveness and safety. J Clin Psychiatry.

[CR68] Gibbons RD, Hur K, Brown CH, Mann JJ (2009). Relationship between antiepileptic drugs and suicide attempts in patients with bipolar disorder. Arch Gen Psychiatry.

[CR69] Gijsman HJ, Geddes JR, Rendell JM, Nolen WA, Goodwin GM (2004). Antidepressants for bipolar depression: systematic review of randomized, controlled trials. Am J Psychiatry.

[CR70] Gitlin MJ (2018). Antidepressants in bipolar depression: an enduring controversy. Int J Bipolar Disord.

[CR71] Goodwin FK, Jamison KR (2007). Manic-depressive illness.

[CR72] Goodwin FK, Fireman B, Simon GE, Hunkeler EM, Lee J, Revicki D (2003). Suicide risk in bipolar disorder during treatment with lithium and divalproex. JAMA.

[CR73] Goodwin GM, Haddad PM, Ferrier IN (2016). Evidence-based guidelines for treating bipolar disorder: revised third edition recommendations from the British Association for Psychopharmacology. J Psychopharmacol..

[CR74] Grady SE, Marsh TA, Tenhouse A, Klein K (2017). Ketamine for the treatment of major depressive disorder and bipolar depression: review of the literature. Ment Health Clin.

[CR75] Grunebaum MF, Ellis SP, Keip JG (2017). Ketamine vs. midazolam in bipolar depression with suicidal thoughts. Bipolar Disord.

[CR76] Halaris A (2017). Inflammation-associated co-morbidity between depression and cardiovascular disease. Curr Top Behav Neurosci.

[CR77] Hällgren J, Ösby U, Westman J, Gissler M (2018). Mortality trends in external causes of death in people with mental health disorders in Sweden, 1987-2010. Scand J Public Health.

[CR78] Hammad TA, Laughren TP, Racoosin JA (2006). Suicide rates in short-term randomized controlled trials of newer antidepressants. J Clin Psychopharmacol.

[CR79] Hjorthøj C, Drivsholm-Østergaard ML, Eriksen-Benros M (2015). Association between alcohol and substance use disorders and all-cause and cause-specific mortality in schizophrenia, bipolar disorder, and unipolar depression: nationwide, prospective, register-based study. Lancet Psychiatry.

[CR80] Isometsä ET (2005). Suicide in bipolar I disorder in Finland: psychological autopsy findings from the National Suicide Prevention Project in Finland. Arch Suicide Res.

[CR81] Itagaki K, Takebayashi M, Shibasaki C (2017). Factors associated with relapse after a response to electroconvulsive therapy in unipolar versus bipolar depression. J Affect Disord.

[CR82] Kessler RC, Berglund P, Borges G, Nock M, Wang PS (2005). Trends in suicide ideation, plans, gestures, and attempts in the US, 1990–1992 to 2001–2003. JAMA.

[CR83] Khan A, Khan SR, Leventhal RM, Brown WA (2001). Symptom reduction and suicide risk among patients treated with placebo in antipsychotic clinical trials: analysis of the FDA database. Am J Psychiatry.

[CR84] Khan A, Khan SR, Hobus J (2011). Differential pattern of response in mood symptoms and suicide risk measures in severely ill depressed patients assigned to citalopram with placebo or citalopram combined with lithium: role of lithium levels. J Psychiatr Res.

[CR85] Koukopoulos A, Reginaldi D, Tondo L, Visioli C, Baldessarini RJ (2013). Course sequences in bipolar disorder: depressions preceding or following manias or hypomanias. J Affect Disord.

[CR86] Kupka RW, Altshuler LL, Nolen WA (2007). Three times more days depressed than manic or hypomanic in both bipolar I and bipolar II disorder. Bipolar Disord.

[CR87] Large M, Swaraj S (2018). Suicide, substance use and natural causes are respectively the most important causes of mortality in the first year post discharge from psychiatric hospitals. Evid Based Ment Health.

[CR88] Laughren TP. Proceedings of a meeting of the Psychopharmacology Drug Advisory Committee (PDAC) concerning suicidal risk in trials of antidepressant drugs in juvenile and adult patients, 2006. http://www.fda.gov/ohrms/dockets/ac/06/briefing//2006-4272b1-01-fda.pdf. Accessed 22 Dec 2018.

[CR89] Lauterbach E, Felber W, Müller-Oerlinghausen B (2008). Adjunctive lithium treatment in the prevention of suicidal behavior in depressive disorders: randomized, placebo-controlled, one-year trial. Acta Psychiatr Scand.

[CR90] Lewitzka U, Bauer M, Felber W, Müller-Oerlinghausen B (2013). Antisuicidal effect of lithium: current state of research and its clinical implications for the long-term treatment of affective disorders. Nervenärzt.

[CR91] Li XB, Tang YL, Wang CY, de Leon J (2015). Clozapine for treatment-resistant bipolar disorder: systematic review. Bipolar Disord.

[CR92] Liu B, Zhang Y, Fang H (2017). Efficacy and safety of long-term antidepressant treatment for bipolar disorders: meta-analysis of randomized controlled trials. J Affect Disord.

[CR93] Liu X, Agerbo E, Li J, Meltzer-Brody S, Bergink V, Munk-Olsen T (2017). Depression and anxiety in the postpartum period and risk of bipolar disorder: Danish nationwide register-based cohort study. J Clin Psychiatry.

[CR94] Lovas DA, Schuman-Olivier Z (2018). Mindfulness-based cognitive therapy for bipolar disorder: systematic review. J Affect Disord.

[CR95] Maj M, Pirozzi R, Magliano L, Fiorillo A, Bartoli L (2006). Agitated, “unipolar” major depression: prevalence, phenomenology and outcome. J Clin Psychiatry.

[CR96] Marangell LB, Dennehy EB, Wisniewski SR (2008). Case-control analyses of the impact of pharmacotherapy on prospectively observed suicide attempts and completed suicides in bipolar disorder. J Clin Psychiatry.

[CR97] Martinez-Botella G, Salituro FG, Harrison BL (2017). Neuroactive steroids: SAGE-217, a clinical next-generation neuroactive steroid positive allosteric modulator of the GABA_A_ receptor. J Med Chem.

[CR98] McCauley E, Berk MS, Asarnow JR (2018). Efficacy of dialectical behavioral therapy for adolescents at high risk for suicide: randomized clinical trial. JAMA Psychiatry.

[CR99] McElroy SL, Weisler RH, Chan W (2010). Double-blind, placebo-controlled study of quetiapine and paroxetine as monotherapy in adults with bipolar depression (EMBOLDEN II). J Clin Psychiatry.

[CR100] McGirr A, Vöhringer PA, Ghaemi SN, Lam RW, Yatham LN (2016). Safety and efficacy of adjunctive second generation antidepressant therapy with a mood-stabilizer or an atypical antipsychotic in acute bipolar depression: systematic review and meta-analysis of randomized placebo-controlled trials. Lancet Psychiatry.

[CR101] McIntyre RS, Soczynska JK, Beyer JL (2007). Medical comorbidity in bipolar disorder: reprioritizing unmet needs. Curr Opin Psychiatry.

[CR102] McMahon K, Herr NR, Zerubavel N, Hoertel N, Neacsiu AD (2016). Psychotherapeutic treatment of bipolar depression. Psychiatr Clin N Am.

[CR103] Meltzer HY, Alphs L, Green AI (2003). Clozapine treatment for suicidality in schizophrenia: international suicide prevention trial (InterSePT). Arch Gen Psychiatry.

[CR104] Messer T, Lammers G, Müller-Siecheneder F, Schmidt RF, Latifi S (2017). Substance abuse in patients with bipolar disorder: systematic review and meta-analysis. Psychiatry Res.

[CR105] Möller HJ (2006). Is there evidence for negative effects of antidepressants on suicidality in depressive patients? Systematic review. Eur Arch Psychiatry Clin Neurosci.

[CR106] Müller-Oerlinghausen B, Ahrens B, Felber W, Bauer M, Grof P, Müller-Oerlinghausen B (2006). Suicide-preventive and mortality-reducing effect of lithium. Lithium in neuropsychiatry.

[CR107] Nemeroff CB, Evans DL, Gyulai L (2001). Double-blind, placebo-controlled comparison of imipramine and paroxetine in the treatment of bipolar depression. Am J Psychiatry.

[CR108] Nierenberg AA, Alpert JE, Gardner-Schuster EE, Seay S, Mischoulon D (2008). Vagus nerve stimulation: 2-year outcomes for bipolar versus unipolar treatment-resistant depression. Biol Psychiatry.

[CR109] Nock MK, Borges G, Bromet EJ (2008). Cross-national prevalence and risk factors for suicidal ideation, plans and attempts. Br J Psychiatry.

[CR110] Olfson M, Wall M, Wang S, Crystal S, Liu SM, Gerhard T, Blanco C (2016). Short-term suicide risk after psychiatric hospital discharge. JAMA Psychiatry.

[CR111] Oquendo MA, Galfalvy HC, Currier D (2011). Treatment of suicide attempters with bipolar disorder: randomized clinical trial comparing lithium and valproate in the prevention of suicidal behavior. Am J Psychiatry.

[CR112] Ösby U, Brandt L, Correia N, Ekbom A, Sparén P (2001). Excess mortality in bipolar and unipolar disorder in Sweden. Arch Gen Psychiatry.

[CR113] Ösby U, Westman J, Hällgren J, Gissler M (2018). Mortality trends in cardiovascular causes in schizophrenia, bipolar and unipolar mood disorder in Sweden 1987–2010. Eur J Pub Health.

[CR114] Pacchiarotti I, Bond DJ, Baldessarini RJ (2013). International Society for Bipolar Disorders (ISBD) Task-Force report on antidepressant use in bipolar disorders. Am J Psychiatry.

[CR115] Parsaik AK, Singh B, Khosh-Chashm D, Mascarenhas SS (2015). Efficacy of ketamine in bipolar depression: systematic review and meta-analysis. J Psychiatr Pract.

[CR116] Pavlova B, Perlis RH, Uher R (2015). Lifetime prevalence er of anxiety disorders in people with bipolar disorder: systematic review and meta-analysis. Lancet Psychiatry.

[CR117] Perugi G, Medda P, Toni C (2017). Role of electroconvulsive therapy (ECT) in bipolar disorder: effectiveness in 522 patients with bipolar depression, mixed-state, mania and catatonic features. Curr Neuropharmacol.

[CR118] Pompili M, Baldessarini RJ, Forte A (2016). Do atypical antipsychotics have antisuicidal effects? A hypothesis-generating overview. Int J Mol Sci.

[CR119] Post RM, Leverich GS, Kupka RW (2010). Early-onset bipolar disorder and treatment delay are risk factors for poor outcome in adulthood. J Clin Psychiatry.

[CR120] Post RM, Leverich GS, McElroy S (2018). Prevalence of Axis-II comorbidities in bipolar disorder: relationship to mood-state. Bipolar Disord.

[CR121] Preti A, Vrublevska J, Veroniki AA, Huedo-Medina TB, Fountoulakis KN (2016). Prevalence, impact and treatment of generalized anxiety disorder in bipolar disorder: systematic review and meta-analysis. Evid Based Ment Health.

[CR122] Ragguett RM, McIntyre RS (2019). Cariprazine for the treatment of bipolar depression: a review. Expert Rev Neurother.

[CR123] Ragguett RM, Rong C, Kratiuk K, McIntyre RS (2019). Rapastinel: investigational NMDA-R modulator for major depressive disorder: evidence to date. Expert Opin Investig Drugs.

[CR124] Reinares M, Rosa AR, Franco C (2013). Systematic review on the role of anticonvulsants in the treatment of acute bipolar depression. Int J Neuropsychopharmacol.

[CR125] Roberts E, Cipriani A, Geddes JR, Nierenberg AA, Young AH (2017). Evidence for lithium in suicide prevention. Br J Psychiatry.

[CR126] Sachs GS, Nierenberg AA, Calabrese JR (2007). Effectiveness of adjunctive antidepressant treatment for bipolar depression. New Engl J Med.

[CR127] Salcedo S, Gold AK, Sheikh S (2016). Empirically supported psychosocial interventions for bipolar disorder: current state of the research. J Affect Disord.

[CR128] Salloum NC, Walker MC, Gangwani S, Conway CR (2017). Emergence of mania in two middle-aged patients with a history of unipolar, treatment-refractory depression receiving vagus nerve stimulation. Bipolar Disord.

[CR129] Sani G, Fiorillo A (2019). Use of lithium in mixed states. CNS Spectr.

[CR130] Saunders KE, Hawton K (2013). Clinical assessment and crisis intervention for the suicidal bipolar disorder patient. Bipolar Disord.

[CR131] Schatzberg AF (2019). A word to the wise about intranasal esketamine. Am J Psychiatry.

[CR132] Schneider-Thoma J, Efthimiou O, Huhn M (2018). Second-generation antipsychotic drugs and short-term mortality: systematic review and meta-analysis of randomized placebo-controlled trials. Lancet Psychiatry.

[CR133] Scott LJ (2019). Brexanolone: first global approval. Drugs.

[CR134] Selle V, Schalkwijk S, Vázquez GH, Baldessarini RJ (2014). Treatments for acute bipolar depression: meta-analyses of placebo-controlled, monotherapy trials of anticonvulsants, lithium, and second-generation antipsychotics. Pharmacopsychiatry.

[CR135] Shelton RC, Stahl SM (2004). Risperidone and paroxetine given singly and in combination for bipolar depression. J Clin Psychiatry.

[CR136] Shen H, Zhang L, Xu C, Zhu J, Chen M, Fang Y (2018). Analysis of misdiagnosis of bipolar disorder in an outpatient setting. Shanghai Arch Psychiatry.

[CR137] Sidor MM, MacQueen GM (2012). Update on antidepressant use in bipolar depression. Curr Psychiatry Rep.

[CR138] Simon RI, Hales RE (2012). Textbook of suicide assessment and management.

[CR139] Smith KA, Cipriani A (2017). Lithium and suicide in mood disorders: updated meta-review on the scientific literature. Bipolar Disord.

[CR140] Solmi M, Veronese N, Zaninoto L (2016). Lamotrigine compared to placebo and other agents with antidepressant activity in patients with unipolar and bipolar depression: comprehensive meta-analysis of efficacy and safety outcomes in short-term trials. CNS Spectr.

[CR141] Song J, Sjolander A, Joas E (2017). Suicidal behavior during lithium and valproate treatment: a within-individual 8-year prospective study of 50,000 patients with bipolar disorder. Am J Psychiatry.

[CR142] Staudt-Hansen P, Frahm-Laursen M, Grøntved S, Puggard-Vogt-Straszek S, Licht RW, Nielsen RE (2019). Increasing mortality gap for patients diagnosed with bipolar disorder: nationwide study with 20 years of follow-up. Bipolar Disord.

[CR143] Stensland MD, Schultz JF, Frytak JR (2008). Diagnosis of unipolar depression following initial identification of bipolar disorder: common and costly misdiagnosis. J Clin Psychiatry.

[CR144] Stokes PRA, Kalk NJ, Young AH (2017). Bipolar disorder and addictions: the elephant in the room. Br J Psychiatry.

[CR145] Suzuki M, Dallaspezia S, Locatelli C, Uchiyama M, Colombo C, Benedetti F (2018). Does early response predict subsequent remission in bipolar depression treated with repeated sleep deprivation combined with light therapy and lithium?. J Affect Disord.

[CR146] Tamayo JM, Zarate CA, Vieta E, Vázquez GH, Tohen M (2010). Level of response and safety of pharmacological monotherapy in the treatment of acute bipolar I disorder phases: systematic review and meta-analysis. Int J Neuropsychopharmacol.

[CR147] Tarsy Daniel, Lungu Codrin, Baldessarini Ross J. (2011). Epidemiology of tardive dyskinesia before and during the era of modern antipsychotic drugs. Handbook of Clinical Neurology.

[CR148] Taylor DM, Cornelius V, Smith L, Young AH (2014). Comparative efficacy and acceptability of drug treatments for bipolar depression: a multiple-treatments meta-analysis. Acta Psychiatr Scand.

[CR149] Thies-Flechtner K, Müller-Oerlinghausen B, Seibert W, Walther A, Greil W (1996). Effect of prophylactic treatment on suicide risk in patients with major affective disorders: data from a randomized prospective trial. Pharmacopsychiatry.

[CR150] Tiihonen J, Wahlbeck K, Lönnqvist J (2006). Effectiveness of antipsychotic treatments in a nationwide cohort of patients in community care after first hospitalization due to schizophrenia and schizoaffective disorder: observational follow-up study. BMJ.

[CR151] Tohen M, Vieta E, Calabrese J (2003). Efficacy of olanzapine and olanzapine-fluoxetine combination in the treatment of bipolar I depression. Arch Gen Psychiatry.

[CR152] Tondo L, Baldessarini RJ, Koslow SH, Ruiz P, Nemeroff CB (2014). Reduction of suicidal behavior in bipolar disorder patients during long-term treatment with lithium. Concise guide to understanding suicide: epidemiology pathophysiology and prevention.

[CR153] Tondo L, Baldessarini RJ, Yildiz A, Nemeroff C, Ruiz P (2015). Chapt 37: Suicide in bipolar disorder. The bipolar book: history, neurobiology, and treatment.

[CR154] Tondo L, Baldessarini RJ (2018). Antisuicidal effects in mood disorders: are they unique to lithium?. Pharmacopsychiatry.

[CR155] Tondo L, Baldessarini RJ, Hennen J, Floris G, Silvetti F, Tohen M (1998). Lithium treatment and risk of suicidal behavior in bipolar disorder patients. J Clin Psychiatry.

[CR156] Tondo L, Baldessarini RJ, Hennen J (1999). Suicide attempts in major affective disorder patients with comorbid substance use disorders. J Clin Psychiatry.

[CR157] Tondo L, Hennen J, Baldessarini RJ (2001). Reduced suicide risk with long-term lithium treatment in major affective illness: meta-analysis. Acta Psychiatr Scand.

[CR158] Tondo L, Lepri B, Baldessarini RJ (2008). Suicidal status during antidepressant treatment in 789 Sardinian patients with major affective disorder. Acta Psychiatr Scand.

[CR159] Tondo L, Vázquez GH, Baldessarini RJ (2010). Mania associated with antidepressant-treatment: comprehensive meta-analytic review. Acta Psychiatr Scand.

[CR160] Tondo L, Baldessarini RJ, Vázquez GH, Lepri B, Visioli C (2013). Clinical responses to antidepressants among 1036 acutely depressed patients with bipolar or unipolar major affective disorders. Acta Psychiatr Scand.

[CR161] Tondo L, Visioli C, Preti A, Baldessarini RJ (2014). Bipolar disorders following initial depression: modeling predictive clinical factors. J Affect Disord.

[CR162] Tondo L, Pompili M, Forte A, Baldessarini RJ (2016). Suicide attempts in bipolar disorders: comprehensive review of 101 reports. Acta Psychiatr Scand.

[CR163] Tondo L, Vázquez GH, Pinna M, Vaccotto PA, Baldessarini RJ (2018). Characteristics of depressive and bipolar patients with mixed features. Acta Psychiatr Scand.

[CR164] Trede K, Salvatore P, Baethge C, Gerhard A, Maggini C, Baldessarini RJ (2005). Manic-depressive illness: evolution in Kraepelin’s textbook, 1883–1926. Harv Rev Psychiatry.

[CR165] Tsai SY, Lee CH, Chen PH (2017). Risk factors for early cardiovascular mortality in patients with bipolar disorder. Psychiatry Clin Neurosci.

[CR166] Tseng PT, Chen YW, Tu KY (2016). Light therapy in the treatment of patients with bipolar depression: meta-analytic study. Eur Neuropsychopharmacol.

[CR167] Undurraga J, Baldessarini RJ, Valenti M (2012). Bipolar depression: clinical correlates of receiving antidepressants. J Affect Disord.

[CR168] Vancampfort D, Vansteelandt K, Correll CU (2013). Metabolic syndrome and metabolic abnormalities in bipolar disorder: meta-analysis of prevalence rates and moderators. Am J Psychiatry.

[CR169] Vázquez GH, Tondo L, Baldessarini RJ (2011). Comparison of antidepressant responses in patients with bipolar vs. unipolar depression: meta-analytic review. Pharmacopsychiatry.

[CR170] Vázquez GH, Tondo L, Undurraga J, Baldessarini RJ (2013). Overview of antidepressant treatment of bipolar depression. Int J Neuropsychopharmacol.

[CR171] Vázquez GH, Tondo L, Undurraga J, Zaratiegui R, Selle V, Baldessarini RJ (2014). Pharmacological treatment of bipolar depression. Adv Psychiatr Treatment.

[CR172] Vázquez GH, Holtzman J, Tondo L, Baldessarini RJ (2015). Efficacy and tolerability of treatments for bipolar depression. J Affect Disord.

[CR173] Vázquez GH, Camino S, Tondo L, Baldessarini RJ (2017). Potential novel treatments for bipolar depression: ketamine, fatty acids, anti-inflammatory agents, and probiotics. CNS Neurol Disord Drug Targets.

[CR174] Vázquez GH, Forte A, Camino S, Tondo L, Baldessarini RJ, Carvalho E, Vieta E (2017). Chapt 17: Psychiatric comorbidity in bipolar disorder: treatment implications: anxiety syndromes and substance abuse. The treatment of bipolar disorder: integrative treatment strategies and future directions.

[CR175] Viktorin A, Lichtenstein P, Thase ME (2014). Risk of switch to mania in patients with bipolar disorder during treatment with an antidepressant alone and in combination with a mood stabilizer. Am J Psychiatry.

[CR176] Vöhringer PA, Perlis RH (2016). Discriminating between bipolar disorder and major depressive disorder. Psychiatr Clin N Am.

[CR177] Wasserman D, Rihmer Z, Rujescu D (2012). European Psychiatric Association (EPA) guidance on suicide treatment and prevention. Eur Psychiatry.

[CR178] WHO (World Health Organization). International suicide rates, 2018. http://www.who.int/gho/mental_health/suicide_rates_crude/en/. Accessed 3 Dec 2018.

[CR179] Widge AS, Malone DA, Dougherty DD (2018). Closing the loop on deep brain stimulation for treatment-resistant depression. Front Neurosci.

[CR180] Wilkinson ST, Sanacora G (2019). A new generation of antidepressants: update on the pharmaceutical pipeline for novel and rapid-acting therapeutics in mood disorders based on glutamate/GABA neurotransmitter systems. Drug Discov Today.

[CR181] Wilkinson ST, Ballard ED, Bloch MH (2018). Effect of a single dose of intravenous ketamine on suicidal ideation: systematic review and individual participant data meta-analysis. Am J Psychiatry.

[CR182] Wu SI, Chen SC, Liu SI (2015). Relative risk of acute myocardial infarction in people with schizophrenia and bipolar disorder: population-based cohort study. PLoS ONE.

[CR183] Yatham LN, Kennedy SH, Parikh SV (2018). Canadian Network for Mood and Anxiety Treatments (CANMAT) and International Society for Bipolar Disorders (ISBD) 2018 guidelines for the management of patients with bipolar disorder. Bipolar Disord.

[CR184] Yerevanian BI, Koek RJ, Mintz J (2007). Bipolar pharmacotherapy and suicidal behavior: lithium, divalproex and carbamazepine. J Affect Disord.

[CR185] Yildiz A, Nemeroff C, Ruiz P (2015). The bipolar book: history, neurobiology, and treatment.

[CR186] Young AH, McElroy SL, Bauer M (2010). Double-blind, placebo-controlled study (EMBOLDEN I) of quietapine and lithium monotherapy in adults in the acute phase of bipolar depression. J Clin Psychiatry.

[CR187] Zalsman G, Hawton K, Wasserman D (2016). Suicide prevention strategies revisited: 10-year systematic review. Lancet Psychiatry.

[CR188] Zimmerman M, Galione JN, Chelminski I, Young D, Dalrymple K, Ruggero CJ (2010). Sustained unemployment in psychiatric outpatients with bipolar disorder: frequency and association with demographic variables and comorbid disorders. Bipolar Disord.

